# Prevalence and serotype distribution of nasopharyngeal carriage of* Streptococcus pneumoniae* among healthy children under 5 years of age in Hainan Province, China

**DOI:** 10.1186/s40249-024-01175-7

**Published:** 2024-01-19

**Authors:** Jian Wang, Li Qiu, Shuang Bai, Wei Zhao, Ao Zhang, Jing Li, Jun-Nan Zhang, Shan-Shan Zhou, Ren Qiu, Zhu Huang, Jv-Xia Liu, Ting-Bin Wang, Xue Sun, Jiang Wu, Qun Zheng, Bin He, Min Lv

**Affiliations:** 1 Beijing Center for Disease Prevention and Control, Beijing, China; 2 Beijing Research Center for Respiratory Infectious Diseases, Beijing, China; 3 Hainan Provincial Center for Disease Control and Prevention, Haikou, China; 4 Haikou Center for Disease Control and Prevention, Haikou, Hainan China; 5 Wanning Center for Disease Control and Prevention, Wanning, Hainan China; 6 Baisha County Center for Disease Control and Prevention, Baisha, Hainan China; 7 Qiongzhong County Center for Disease Control and Prevention, Qiongzhong, Hainan China

**Keywords:** Pneumococcus, Nasopharyngeal carriage, Serotypes distribution, Children, Hainan Province, China

## Abstract

**Background:**

The thirteen-valent pneumococcal conjugate vaccine (PCV13) is not included in the national immunization program and is administered voluntarily with informed consent in China. In preparation for assessing the impact of pilot introduction in Hainan Province, we conducted a carriage study among children under 5 years of age from four locations in Hainan Province, China.

**Methods:**

From March to June 2022, nasopharyngeal (NP) swabs, collected from healthy children aged younger than 59 months who lived in the 4 different locations (Haikou, Wanning, Baisha and Qiongzhong) in Hainan Province, were tested for pneumococcus using conventional culture. Pneumococcal isolates were serotyped using the Quellung reaction. Risk factors associated with pneumococcal colonization were assessed using univariate analysis and multivariable logistic regression adjusting for age, daycare attendance and other factors.

**Results:**

Pneumococcus was isolated in 710 (30.4%) of the 2333 children enrolled. Of 737 pneumococci, 29 serotypes were identified; 60.9% were PCV13 serotypes; the most common vaccine serotypes were 6B (20.4%), 19F (13.0%), 6A (11.9%) and 23F (6.1%); and the most common nonvaccine serotypes were 23A (12.9%), 34 (6.1%) and nontypeable (NT) pneumococci (5.6%). Children vaccinated with PCV13 had lower carriage (17.7% vs 32.5%; *P* = 0.0001) and fewer PCV13 serotypes (41.9% vs 62.7%; *P* = 0.0017) compared to unimmunized children. After adjustment, NP carriage was higher among children attending daycare (a*OR* = 2.3, 95% *CI:* 1.7–3.2), living in rural areas (a*OR* = 1.4, 95% *CI:* 1.1–1.8), living with siblings (a*OR* = 1.3, 95% *CI:* 1.0–1.6) and whose mothers had completed senior high/technical secondary school (a*OR* = 1.5, 95% *CI:* 1.1–2.0). In contrast, completion of 3*–*4 doses of PCV13 were associated with a lower carriage rate (a*OR* = 0.6, 95% *CI:* 0.4–0.9).

**Conclusions:**

We established the baseline of pneumococcal carriage, serotype distribution and PCV13 immunization rates among healthy children under 5 years of age in Hainan Province, prior to the introduction of PCV13 into the national immunization program. The high proportion of PCV13 serotypes suggests that PCV13 introduction will likely have a substantial impact on pneumococcal carriage in Hainan Province.

**Graphical Abstract:**

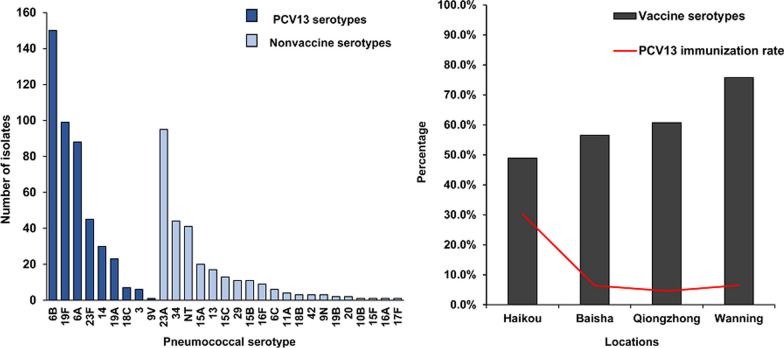

**Supplementary Information:**

The online version contains supplementary material available at 10.1186/s40249-024-01175-7.

## Background

*Streptococcus pneumoniae* (*Spn*) is the leading cause of pneumonia, meningitis, and other serious infections in children and is associated with the greatest number of deaths among children under 5 years [1, 2]. Globally, the implementation of pneumococcal conjugate vaccines (PCVs) has substantially reduced invasive pneumococcal disease (IPD) and pneumonia caused by vaccine serotype (VT) pneumococci [3]. The World Health Organization recommends the inclusion of PCVs in childhood immunization programs worldwide [4].

Nasopharyngeal (NP) carriage of pneumococci is usually asymptomatic and is a prerequisite for the occurrence of pneumococcal mucosal infection, invasive infection and transmission. Young children are more commonly colonized with *Spn* than older children or adults and are a major source of pneumococcal transmission to persons of all ages. Studies have shown that the implementation of PCVs in national immunization programs has significantly reduced NP carriage of vaccine serotypes [5, 6]. Consequently, young children who receive PCVs indirectly protect vaccinated and unvaccinated individuals (including adults) by reducing the prevalence of carriage of vaccine serotypes [7, 8]. Thus, the changes in pneumococcal carriage rates and serotype distribution before and after pneumococcal vaccination are a key biological link to evaluate vaccine efficacy.

China has not established a national or population-wide disease surveillance system for pneumococcal disease and is unable to carry out surveillance of IPD, as exemplified by the Centers for Disease Control in the USA and European countries [9, 10]. Therefore, assessing pneumococcal carriage among children provides a simple and practical approach to evaluate the potential disease burden and vaccine effect in China. Furthermore, the thirteen-valent pneumococcal conjugate vaccine (PCV13) has been licensed for optional use since 2016 and has not been included in the national immunization program [11]. Moreover, there is a lack of baseline data on NP carriage nationwide and across all age groups. Therefore, it is essential to conduct carriage studies among multiage groups of the vulnerable population before the wide use of PCV13 across China, as this can provide crucial data for vaccine evaluation.

This study aimed to analyse the prevalence and serotype distribution of NP carriage among children under 5 years of age from 4 different locations of Hainan Province. These analyses are expected to provide epidemiological baseline data on pneumococcal carriage in Hainan Province, before the introduction of PCVs into the national immunization schedules.

## Methods

### Study setting

Hainan Province is located in the southernmost part of the Chinese mainland, where the annual average temperature is 22*–*27 °C and rainfall is abundant. By the end of 2020, there were 10.12 million residents, of which the nonregistered population was less than 6%. The per capita GDP [Chinese Yuan (CNY) 55,131] and per capita disposable income of households (CNY 27,904) are lower than the national average [12, 13]. PCV13 is not included in the immunization program in China. Completing a four-dose series requires an out-of-pocket expenditure of CNY 1900–2800. In less economically developed regions, the IPD burden is higher due to the natural environment and weaker health care conditions [14], whereas PCV13 coverage is lower which may be related to high cost [15]. Unbalanced regional development and disparities in health care between different areas can also be found in Hainan Province [13].

### Study design

This was a cross-sectional study conducted from March to June 2022. Using data from other carriage studies conducted in China [16], we determined that enrolling 2250 children in 5 age groups (< 12 months, 12*–*23 months, 24*–*35 months, 36*–*47 months and 48*–*59 months, 450 subjects per age group) would enable the detection of 25% carriage of pneumococci. According to the latest census data [13], the ratio of the urban to rural population in Hainan is approximately 6 to 4. Therefore, we planned to enroll 60% of the sample from urban areas and 40% from rural areas. A total of 4 locations (Haikou, Wanning, Baisha and Qiongzhong) were sampled from 18 areas stratified by urban‒rural population proportions and income levels (Additional file 1: Table S1).

In China, points of vaccination (POVs) carry out regular health management and establish vaccination files for children aged 0*–*6 years in community health service centers. POV staff can use these files to recruit respondents. Children visiting POVs are likely to be healthy. In this study, we recruited children from 33 POVs (13 from Haikou, 8 from Wanning, 6 from Qiongzhong and 6 from Baisha, respectively), accounting for 16.9% of the total number of local POVs.

### Study population

Study participants were healthy children aged younger than 59 months who lived in the 4 different locations of Hainan Province. Children were excluded if they had upper or lower respiratory illness or a documented febrile episode within the last 24 h (axillary temperature of ≥ 37.3 °C), if they had congenital malformation or injury of the nasopharynx that would prevent the taking of an NP swab. The parents or guardians of the participants were interviewed regarding demographics, epidemiological factors, vaccination history related to pneumococcus, and risk factors that have been associated with pneumococcal carriage.

### NP sample collection

We followed the previously published recipe and method for the preparation of skim milk-tryptone-glucose-glycerin (STGG) medium [17]. STGG vials were brought to room temperature and vortexed immediately at high speed for 10*–*20 s before being used. An NP specimen was obtained at the clinics by otolaryngologists from each participant using a flexible mini tip size nylon swab (Copan Diagnostics, Inc., Italy). The swab was inserted to the bottom of the STGG medium, and the handle of the swab was cut off using sterile scissors, leaving the tip immersed in 1 ml STGG medium, and the cap was secured. The vial was vortexed for 10*–*20 s to disperse the organisms from the swab. These NP swabs in STGG medium were placed on dry ice within 30 min, transferred to the local microbiology laboratory within 6 h and stored in a − 80 °C refrigerator. After collection, all NP-STGG specimens were transported to the Beijing Centers for Disease Prevention and Control (CDC) on dry ice, and kept at − 80 °C until they were processed.

### Laboratory methods

NP-STGG specimens were thawed at 37 °C and vortexed for approximately 10–20 s. For each NP specimen, 400 µl of STGG media was transferred to 6.0 ml enrichment broth (5 ml of Todd Hewitt broth containing 0.5% yeast extract to which 1 ml rabbit serum was added) and the broth was incubated in 5% carbon dioxide at 37 °C for 7–8 h. Enriched culture (10 µl) was plated on 5% sheep blood agar with 10 mg/l polymyxin B and nalidixic acid, and incubated in 5% carbon dioxide at 37 °C for 18–20 h. Two or more (for each different morphology type) typical pneumococcal colonies were selected and sub-cultured on 5% sheep blood agar. Pneumococci were identified by optochin susceptibility and bile solubility tests. Pneumococcal isolates were serotyped by performing the Quellung reaction with antisera from the Staten Serum Institute (Copenhagen, Denmark).

### Statistical analysis

ANOVA was used to calculate and compare mean age among different locations. The Chi-square test was used for intergroup comparisons of categorical variables such as NP carriage and gender proportions. To evaluate potential risk factors for NP carriage, first univariate analysis using Chi square was performed to identify potential factors associated with carriage. Variables that were significant (*P* < 0.05) in the univariate analysis were further tested by multiple logistic regression using the forward procedure to assess contribution to carriage. *P* < 0.05 was considered statistically significant. Analyses were performed using SPSS version 21.0 (IBM Corp., Armonk, NY, USA) and WPS Office.

## Results

### Population and pneumococcal colonization

A total of 2333 children were enrolled from 4 different locations in this study: 764 from Haikou, 645 from Wanning, 450 from Baisha and 474 from Qiongzhong (Table 1). Of those, 443–508 children were in each age group. Pneumococcus was isolated from 710 (30.4%) children: 213 from Haikou, 221 from Wanning, 145 from Baisha and 131 from Qiongzhong, and the carriage rates were 27.9%, 34.3%, 32.2% and 27.6%, respectively.

**Table 1:** Demographic characteristics of healthy children in Hainan province

Variable	Haikou (*n* = 764)	Wanning (*n* = 645)	Baisha (*n* = 450)	Qiongzhong (*n* = 474)	Total (*n* = 2333)	*P* value
Age (months), median (*IQR*)	29 (14–45)	32 (16–48)	27 (16–45)	29 (14–45)	30 (15–46)	0.1107
Age group, *n*						
< 12 months	148	109	88	98	443	0.5802
12–23 months	156	125	91	95	467	
24–35 months	151	118	93	93	455	
36–47 months	152	126	88	94	460	
≥ 48 months^a^	157	167	90	94	508	
Gender, *n*						
Male	430	355	232	242	1259	0.2019
Female	334	290	218	232	1074	
Number of swabs positive for pneumococcus, *n* (%)	213 (27.9)	221 (34.3)	145 (32.2)	131 (27.6)	710 (30.4)	**0.0261**
Percentage of PCV13 immunization, *n* (%)	233(30.5)	42(6.5)	30(6.7)	22(4.6)	327(14.0)	**< 0.001**
1 dose	41	16	7	11	75	
2 doses	29	12	10	6	57	
3 doses	63	4	9	4	80	
4 doses	100	10	4	1	115	

*IQR* interquartile range, *PCV13* 13-valent pneumococcal conjugate vaccine

^a^Sixty children aged ≥ 60 months. Statistically significant differences (*P* ＜ 0.05) are shown in bold

### Serotype distribution and vaccination coverage

**Fig. 1:**
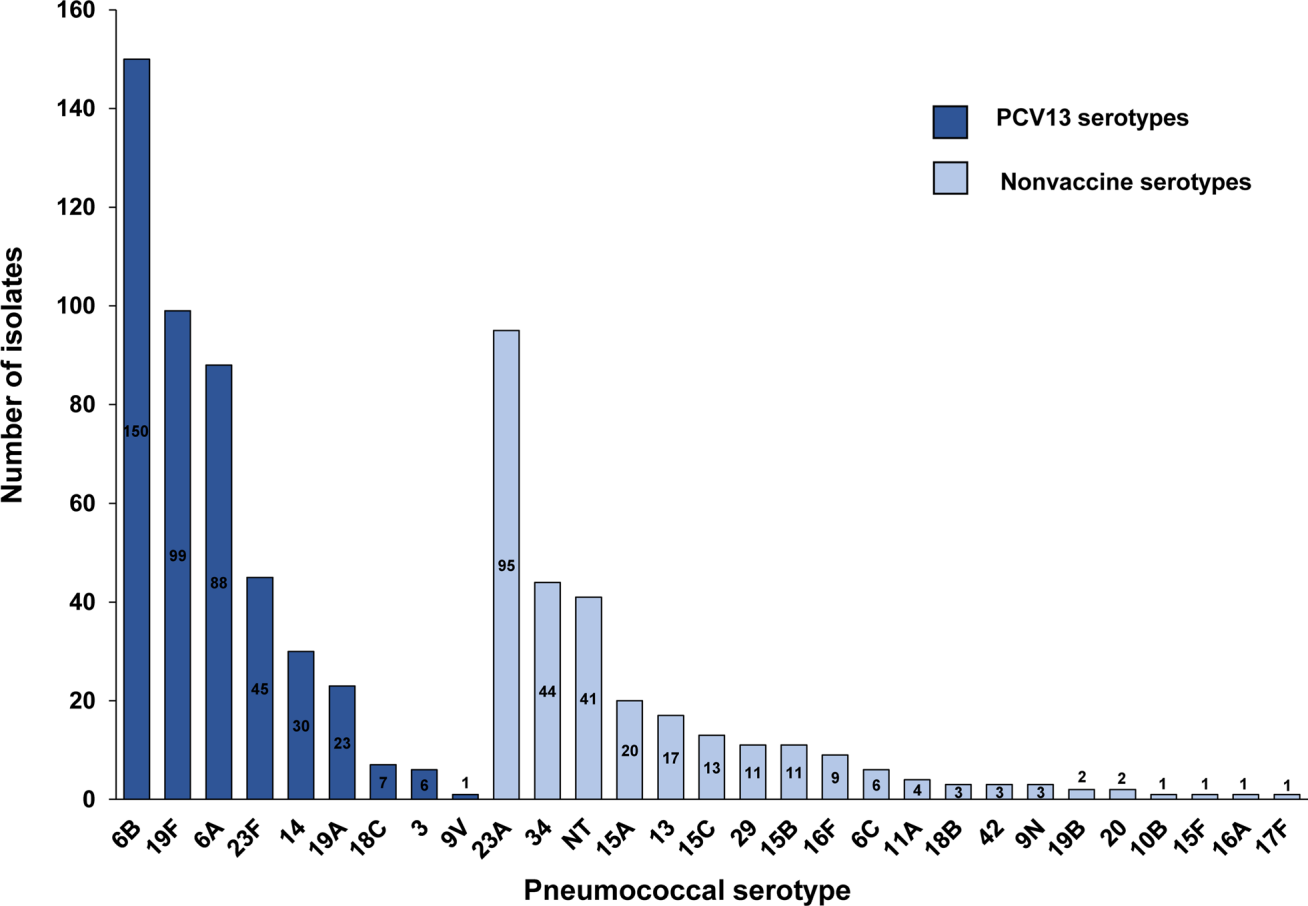
Serotype distribution of pneumococci isolated from Hainan Province among children under 5 years of age (*n* = 737). *NT* nontypeable serotype, *PCV13* 13-valent pneumococcal conjugate vaccine

Twenty-seven children had 2 serotypes isolated from a single specimen, for a total of 737 pneumococcal isolates. Among those pneumococcal isolates, 29 serotypes were identified and the coverage rate of PCV13 was 60.9% (449/737) (Fig. 1). The most common vaccine serotypes were 6B (*n* = 150, 20.4%), 19F (*n* = 99, 13.0%), 6A (*n* = 88, 11.9%) and 23F (*n* = 45, 6.1%). Four vaccine serotypes (1, 4, 5 and 7F) were not isolated from any children, and vaccine serotype 9V was isolated from only 1 child. The most common nonvaccine serotypes were 23A (*n* = 95, 12.9%), 34 (*n* = 44, 6.1%) and nontypeable (NT) pneumococci (*n* = 41, 5.6%).

### PCV13 usage and pneumococcal colonization

Among all the participants, 327 (14.0%) children (PCV-13 group) completed 1–4 doses of PCV13 and 2006 (86.0%) children (non-PCV13 group) did not receive PCV13 or received 1 dose of 23-valent pneumococcal polysaccharide vaccine (PPV23). There was a statistically significant difference in carriage between the two groups for both carriage and PCV13 serotype coverage rates. We compared the prevalence of serotype colonization between the two groups. Pneumococcus was isolated in 58 children (17.7%) and 652 children (32.5%) in the PCV13 and non-PCV13 groups, respectively (17.7% vs 32.5%, *P* = 0.0001). A total of 62 and 675 pneumococcal isolates were identified, and the coverage rates of PCV13 were 41.9% (26/62) and 62.7% (423/675), respectively, in the PCV13 and non-PCV13 groups (41.9% vs 62.7%, *P* = 0.0017). The serotype distribution was similar in the two groups. The most common vaccine serotypes were 6B, 19F and 6A, and the most common nonvaccine serotypes were 23A, 34 and NT pneumococcus.

**Fig. 2:**
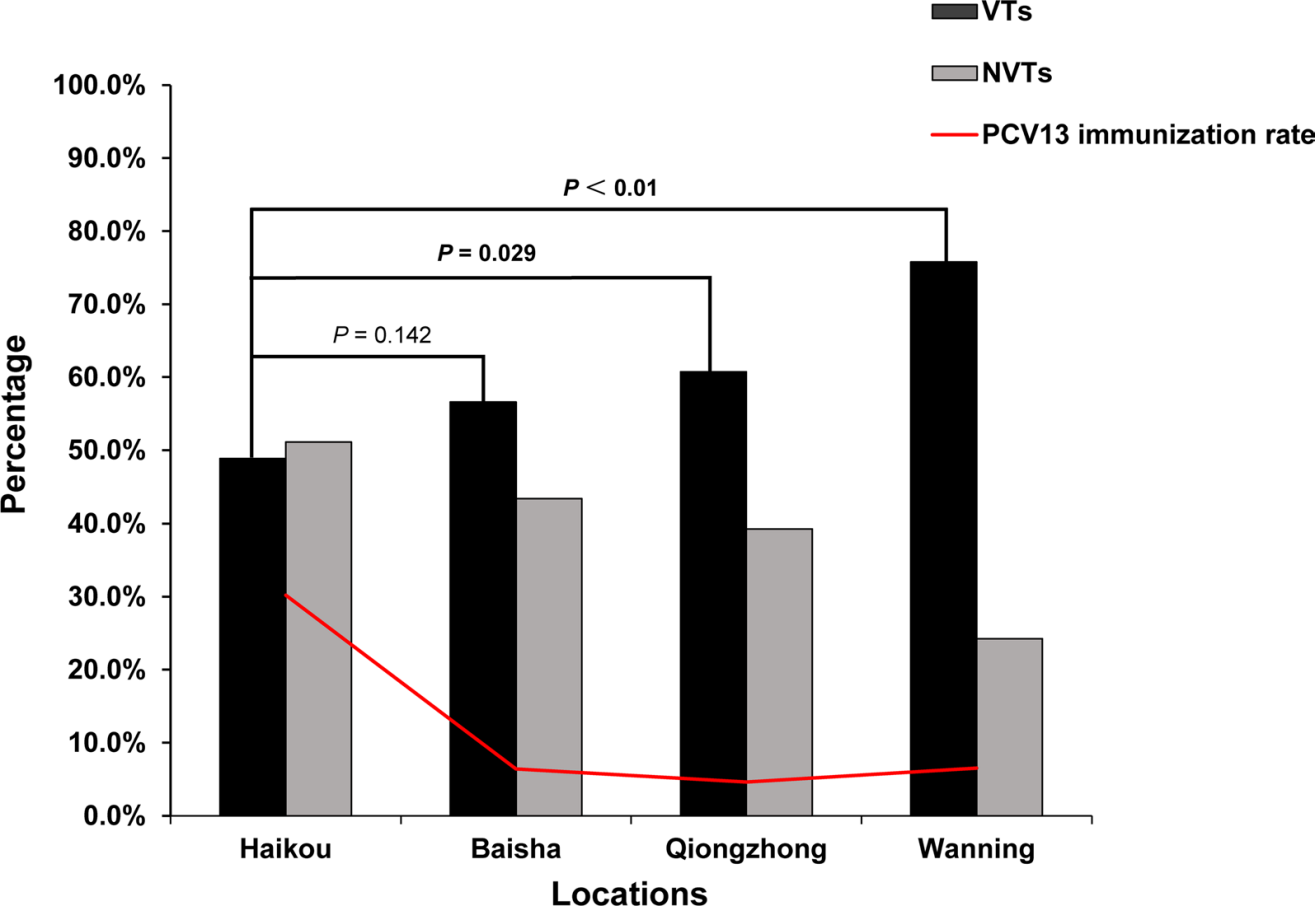
The coverage rate of PCV13 vaccine and nonvaccine serotypes in the 4 locations of Hainan Province. *VTs* vaccine serotypes, *NVTs* nonvaccine serotypes, *PCV13* 13-valent pneumococcal conjugate vaccine

As a comparison, we also evaluated at carriage patterns in the 4 different locations. The proportions of PCV-13-vaccinated children were 30.2% (231/767), 6.5% (42/645), 6.4% (29/450), and 4.6% (22/474) in Haikou, Wanning, Baisha and Qiongzhong, respectively. Haikou, with the highest vaccination rate, had significantly lower PCV13 serotype coverage than Wanning (48.9% vs 75.8%, *P* < 0.01) and Qiongzhong (48.9% vs 60.7%, *P* = 0.029) (Fig. 2). The serotype distribution patterns were also similar in the 4 locations (Additional file 1: Table S2). Notably, 23A was the most common serotype among all isolates identified in Haikou.

### Risk factors associated with pneumococcal colonization

Seven factors, including age, daycare attendance, presence of siblings, residential area, educational level of parents, per capita monthly disposable income and PCV13 vaccination history, were associated with NP carriage (*P* < 0.05 for all) (Table 2). Factors that remained associated with NP carriage after forward elimination included the following: age, daycare attendance, presence of siblings, residential area, educational level of parents, per capita monthly disposable income and PCV13 vaccination history. After adjustment for these factors, NP carriage was higher among children attending daycare (a*OR* = 2.3, 95% *CI:* 1.7–3,2), living in rural areas (a*OR* = 1.4, 95% *CI:* 1.1–1.8), living with siblings (a*OR* = 1.3, 95% *CI:* 1.0–1.6) and whose mothers had completed senior high/technical secondary school (a*OR* = 1.5, 95% *CI:* 1.1–2.0) (Table 3). In contrast, for PCV13 vaccination history, only completion of 3–4 doses of PCV13 was associated with a lower likelihood of NP carriage (a*OR* = 0.6, 95% *CI:* 0.4–0.9, *P* = 0.032).

**Table 2:** Univariate analysis of risk factors for pneumococcal carriage

Epidemiologic factors	Pneumococcus-positive^a^, *n* (%)	Pneumococcus-negative^b^, *n* (%)	*P* value
Demographics			
Male gender	379 (53.4)	880 (54.2)	0.718
Age, median (*IQR*)	38 (20–50)	26 (13–44)	**< 0.001**
< 12 months	94 (13.2)	349 (21.5)	
12–23 months	117 (16.5)	350 (21.6)	
24–35 months	112 (15.8)	343 (21.1)	
36–47 months	184 (25.9)	276 (17.0)	
≥ 48 months	203 (28.6)	305 (18.8)	
Gestational age			0.238
Premature	33 (4.7)	96 (5.9)	
Postmature	677 (95.3)	1527 (94.1)	
Birth weight			0.943
< 2.5 kg	50 (7.0)	108 (6.6)	
2.5 kg– < 4 kg	643 (90.6)	1476 (91.0)	
≥ 4 kg	17 (2.4)	39 (2.4)	
Maternal age at delivery			0.088
< 35 years	618 (87.0)	1368 (84.3)	
≥ 35 years	92 (13.0)	255 (15.71)	
Household registration			0.549
Local	612 (86.2)	1415 (87.2)	
Non-local	98 (13.8)	208 (12.8)	
Breastfeeding (age ≤ 24 months)			0.322
Never breastfed	32 (14.0)	117 (15.7)	
Weaned	146 (64.1)	437 (58.5)	
Still breastfed	50 (21.9)	193 (24.8)	
Daycare attendance			**< 0.001**
No	345 (48.6)	1135 (69.9)	
Yes	365 (51.4)	488 (30.1)	
Presence of siblings			**< 0.001**
No	202 (28.5)	623 (38.4)	
Yes	508 (71.5)	1000 (61.6)	
Household characteristics			
Household tobacco exposure			0.961
No	208 (29.3)	479 (29.5)	
Yes	502 (70.9)	1144 (70.5)	
Average living space			0.085
< 10 m^2^	41 (5.8)	62 (3.8)	
10–20 m^2^	302 (42.5)	729 (44.9)	
> 20 m^2^	367 (51.7)	832 (51.3)	
Residential area			**< 0.001**
Urban area	202 (28.5)	685 (42.2)	
Rural area	508 (71.5)	938 (57.8)	
Socioeconomic status			
Educational level of mother			**< 0.001**
Elementary/Junior high school	349 (49.2)	677 (41.7)	
Senior high/Technical secondary school	192 (27.0)	371 (22.9)	
Junior college/Bachelor/Master	169 (23.8)	575 (35.4)	
Educational level of father			**< 0.001**
Elementary/Junior high school	328 (46.2)	621 (38.3)	
Senior high/Technical secondary school	191 (26.9)	434 (26.7)	
Junior college/Bachelor/Master	191 (26.9)	568 (35.0)	
Per capita monthly disposable income			**< 0.001**
< 3000 CNY (415 USD)	288 (40.6)	572 (35.2)	
3000–5999 CNY (415–831 USD)	289 (40.7)	604 (37.2)	
≥ 6000 CNY (831 USD)	133 (18.7)	447 (27.6)	
Vaccination history			
PCV13			**< 0.001**
0 dose	652 (91.8)	1354 (83.4)	
1–2 doses	28 (4.00)	167 (10.3)	
≥ 3 doses	30 (4.2)	102 (6.3)	
Hib vaccines^c^			0.155
Yes	483 (68.0)	1054 (64.9)	
No	227 (32.0)	569 (35.1)	
Influenza vaccines			0.503
Yes	572 (80.6)	1286 (79.2)	
No	138 (19.4)	337 (20.8)	
Antimicrobial use			
Antibiotics within 10 days prior to sampling			0.123
Yes	681 (95.9)	1578 (97.2)	
No	29 (4.1)	45 (2.8)	

*IQR* interquartile range, *CNY* Chinese Yuan, *USD* US dollar, *PCV13* 13-valent pneumococcal conjugate vaccine, *Hib Haemophilus influenza*e type b

^a^Pneumococcus-positive means culture positive for pneumococcus

^b^Pneumococcus-negative means culture negative for pneumococcus

^c^All vaccines containing Hib components. Statistically significant differences (*P* ＜ 0.05) are shown in bold

**Table 3:** Multivariate analysis of risk factors for pneumococcal carriage

Epidemiologic factors	*OR* (95% *CI*)	*P* value	a*OR* (95% *CI*)	*P* value
Demographics				
Age				
< 12 months	Reference			
12–23 months	1.2 (0.9–1.7)	0.171	1.3 (0.9–1.7)	0.167
24–35 months	1.2 (0.9–1.7)	0.227	1.1 (0.8–1.5)	0.685
36–47 months	2.5 (1.8–3.3)	**< 0.001**	1.3 (0.9–1.9)	0.235
≥ 48 months	2.5 (1.9–3.3)	**< 0.001**	1.0 (0.7–1.5)	0.988
Daycare attendance				
No	Reference			
Yes	2.5 (2.1–3.0)	**< 0.001**	2.3 (1.7–3.2)	**< 0.001**
Presence of siblings				
No	Reference			
Yes	1.6 (1.3–1.9)	**< 0.001**	1.3 (1.0–1.6)	**0.024**
Household characteristics				
Residential area				
Urban area	Reference			
Rural area	1.8 (1.5–2.2)	**< 0.001**	1.4 (1.1–1.8)	**0.003**
Socioeconomic status				
Educational level of mother				
Elementary/Junior high school	1.8 (1.4–2.2)	**< 0.001**	1.3 (1.0–1.8)	0.085
Senior high/Technical secondary school	1.8 (1.4–2.3)	**< 0.001**	1.5 (1.1–2.0)	**0.007**
Junior college/Bachelor/Master	Reference			
Educational level of father				
Elementary/Junior high school	1.6 (1.3–1.9)	**< 0.001**	1.0 (0.7–1.4)	0.996
Senior high/Technical secondary school	1.3 (1.0–1.7)	**0.026**	0.9 (0.6–1.1)	0.273
Junior college/Bachelor/Master	Reference			
Per capita monthly disposable income				
< 3000 CNY (415 USD)	1.7 (1.3–2.2)	** < 0.001**	1.3 (1.0–1.7)	0.083
3000–5999 CNY (415–831 USD)	1.6 (1.3–2.0)	**< 0.001**	1.3 (1.0–1.6)	0.070
≥ 6000 CNY (831 USD)	Reference			
Vaccination history				
PCV13				
0 dose	Reference			
1–2 doses	0.7 (0.5–1.0)	**0.029**	0.9 (0.6–1.4)	0.488
≥ 3 doses	0.4 (0.2–0.5)	**< 0.001**	0.6 (0.4–0.9)	**0.032**

Reference group is participants without this characteristic. Statistically significant differences (*P* ＜ 0.05) are shown in bold

*OR* odds ratio, *aOR* adjusted odds ratio, *CI* confidence interval, *CNY* Chinese Yuan, *USD* US dollar, *PCV13* 13-valent pneumococcal conjugate vaccine

## Discussion

To our knowledge, this cross-sectional study is the largest NP carriage investigation among healthy children in Hainan Province. We present the results of a population-based survey on NP carriage prior to adopting PCVs in China’s national immunization schedule. The baseline data will enable the estimation of the vaccine impact of PCV13 implementation.

The overall NP carriage rate was 30.4% in our study. Previous surveillance studies of NP carriage, prior to the introduction of PCV13, revealed a higher rate of NP carriage among children under 5 years of age in some Asian countries. In Thailand, Indonesia and India, the overall carriage rates were 35.9%, 49.5% and 54.5%, respectively [18–20]. A meta-analysis of studies conducted in Southeast Asia showed that the pooled prevalence of NP carriage among healthy children under 5 years of age was 36.0% (95% *CI:* 34.2–37.8%) [21]. The overall NP carriage rate in our study was higher than that reported from other places in China. Earlier studies of NP carriage in Beijing, Shanghai and Chongqing indicated that the overall carriage rates were 22.0%, 16.6% and 16.6%, respectively [16, 22, 23]. In 2017, prior to the introduction of PCV13 in China, a meta-analysis of data from young children found that the pooled prevalence of NP carriage was 21.4% (95% *CI:* 18.3–24.4%) [24].

Several factors could account for the discrepancy observed in our study, including geography, socioeconomic status, sample collection and differences in laboratory procedures used for *Spn* identification. For example, higher NP carriage rates were observed in less-developed countries [25, 26]. In the sample collection and processing procedures, we ensured that the NP swabs were placed in STGG medium on dry ice as quickly as possible (within 30 min). This allowed for storage and transport of NP specimens at temperatures below − 70 °C, which proved to be optimal conditions without loss of colony-forming units [17]. In addition, we used a broth enrichment step in our laboratory procedures, which was useful for the detection of pneumococci in culture, especially for samples with low organism concentrations [27].

The top 5 serotypes in our study (6B, 19F, 23A, 6A, and 23F) accounted for 64.7% of all carriage strains. In line with other carriage studies and systematic reviews conducted in China [16, 22, 24, 28], vaccine serotypes 6B, 19F, 6A and 23F were highly prevalent; other vaccine serotypes 1, 4, 5, 7F and 9V were relatively rare in our study. Notably, the serotype distribution was relatively consistent with the serotypes frequently associated with IPD in China [29]. Of substantial concern was the high prevalence of certain nonvaccine serotypes, which could reduce the benefits of vaccination. The top 3 nonvaccine serotypes (23A, 34 and NT) accounted for 24.4% of all carriage strains. Serotype 23A was prevalent in our study, particularly in Haikou, where it was the dominant serotype. In contrast, it was rarely observed in other carriage studies conducted in different places of China and other countries. Considering the relatively high PCV13 immunization rate in Haikou, the potential risk of serotype replacement by nonvaccine serotypes requires vigilance. Throughout Asia and Australia [18, 19, 30, 31], NT pneumococci are the most commonly isolated organisms, with some NT pneumococci being co-colonizing isolates. Our findings also revealed that some NT pneumococci co-colonized with other serotypes. Although NT pneumococci are infrequent causes of invasive disease in young children, they are associated with a variety of mucosal diseases and may serve as an essential reservoir for antimicrobial resistance genes [31].

The NP carriage rate and serotype distribution varied by geography and were altered by the implementation of PCVs and socioeconomic status. We recruited healthy children from 4 different locations in Hainan Province. Haikou, the provincial capital with a higher economic level than other locations, had the highest number of children vaccinated with PCV13. Consequently, the lowest PCV13 serotype coverage rates were also observed in Haikou. Although the overall carriage rates remained stable or moderately declined after the introduction of PCVs in several studies [32–34], lower carriage rates were found among children vaccinated with PCV13 in our study. This could be attributed to higher economic levels and better living conditions, which reduce the likelihood of pneumococcal carriage. In addition, since PCV13 is not widely used in China, nonvaccine serotype replacement was less obvious than that in other countries with high PCV13 vaccination coverage.

Several expected epidemiologic factors showed associations with pneumococcal carriage. Consistent with previous findings [25, 33, 35], factors such as age, daycare attendance, the presence of siblings, rural residence and lower socioeconomic status were significantly associated with higher rates of pneumococcal colonization. Previous studies reported that breastfeeding was associated with lower rates of pneumococcal colonization [36, 37]. Considering that the majority of Chinese children are weaned between 1 and 2 years of age and that breastfeeding has minimal impact on pneumococcal colonization in children over 2 years of age, children under 2 years were included in the bivariate analysis. However, in our study, breastfeeding showed no relationship to colonization. Antibiotic therapy, previously associated with reduced odds of pneumococcal colonization [33], showed no such association in our study. After adjustment for multiple factors, only 5 factors remained statistically significant. Consistent with several previous studies [33, 35], having siblings and daycare attendance were identified as risk factors for carriage. This is likely due to the transmission of pneumococci between children within the same family and kindergarten through close contact. Studies conducted in the UK have suggested that reduced-dose schedules are immunogenic and have little impact on IPD or pneumococcal community-acquired pneumonia cases [38, 39]. However, 2 primary doses of the PCVs received in the first year of life had a weak effect on colonization. In line with the study, we found that completion of 3–4 doses of PCV13 was associated with a lower likelihood of NP carriage[7]. Several factors could explain this observation. First, echoing previous findings, high IgG concentrations are required to reduce and prevent NP carriage [40]. After the infant series (3 doses of PCV13) and the toddler dose (4 doses of PCV13), children acquired high IgG concentrations, contributing to the clearance of NP carriage and prevention of new colonization. 

Our study has several limitations. First, our investigation was a single-center study confined to Hainan Province; thus, the findings might not represent the overall NP carriage trends in China. Conducting a multicenter investigation of NP carriage and serotype distribution is essential to provide important epidemiological baseline data for assessing the impact of PCVs on NP carriage, especially prior to the introduction of PCV13 into the national immunization program. Second, carriage patterns also varied with seasonality. Our investigation took place over a 1-month period in each location, and some vaccine serotypes were not found or were uncommon in our study; these serotypes are common causes of IPD. Therefore, the NP carriage patterns may not accurately predict the distribution of specific serotypes that cause severe disease in the local area.

## Conclusions

This study provides baseline pneumococcal carriage, serotype distribution and PCV13 immunization rates among healthy children under 5 years of age in Hainan Province prior to the introduction of PCV13 into the national immunization program. Our results suggest that given the high coverage of PCV13 serotypes, the introduction of PCVs into the national immunization program will likely have a substantial impact on pneumococcal carriage in Hainan Province. Children vulnerable to pneumococcal disease would benefit from the use of PCVs. Due to the diversity of pneumococcal serotypes, it is essential to perform future longitudinal and multicenter surveillance of NP carriage to monitor the effectiveness of current PCVs.

### Supplementary Information

**Additional file 1: Table S1.** Urban–rural population proportions and income levels. **Table S2.** Serotypes distribution of the carriage pneumococcal isolates in the 4 regions of Hainan province.

## Data Availability

All data generated or analysed during this study are included in this published article and its supplementary information files.

## Abbreviations

PCV13: 13-Valent pneumococcal conjugate vaccine
NP: Nasopharyngeal
NT: Nontypeable
*Spn*: *Streptococcus pneumonia*
IPD: Invasive pneumococcal disease
VTs: Vaccine serotypes
NVTs: Non-vaccine serotype
CNY: Chinese Yuan
POV: Point of vaccinations
STGG: Skim milk-tryptone-glucose-glycerin
IQR: Interquartile range
*OR*: Odds ratio
a*OR*: Adjusted odds ratio
*CI*: Confidence interval
