# Supplementary material for: Prevalence and serotype distribution of nasopharyngeal carriage of Streptococcus pneumoniae among healthy children under 5 years of age in Hainan Province, China
Source: Infect Dis Poverty. 2024 Jan 19;13:7. doi: 10.1186/s40249-024-01175-7 (PMC10797996; doi:10.1186/s40249-024-01175-7)
Supplement: Supplementary file 1 — Additional file 1: Table S1. Urban–rural population proportions and income levels. Table S2. Serotypes distribution of the carriage pneumococcal isolates in the 4 regions of Hainan province. [file 40249_2024_1175_MOESM1_ESM.doc]

| **Table S1.** Urban-rural population proportions and income levels | | | | |
| --- | --- | --- | --- | --- |
| Region | Population[1]  (10,000 persons) | Urban population[1] (10,000 persons) | Rural population[1] (10,000 persons) | per capita disposable income of households[2] (CNY) |
| Haikou | 288.7 | 236.0 | 52.7 | 35,025 |
| Wanning | 54.9 | 22.7 | 32.2 | 25,960 |
| Qiongzhong | 18.0 | 5.5 | 12.5 | 21,064 |
| Baisha | 16.4 | 3.3 | 13.2 | 20,206 |
| 1. Population of usual residents at year-end by region (2020). Hainan Statistical Yearbook 2021. https://www.hainan.gov.cn/hainan/tjnj/202111/3fb200cffe374fd5b163da14c2adab81.shtml | | | | |
| 1. Per capita disposable income of households by region (2020). Hainan Statistical Yearbook 2021. https://www.hainan.gov.cn/hainan/tjnj/202111/3fb200cffe374fd5b163da14c2adab81.shtml | | | | |

**Table S2.** Serotypes distribution of the carriage pneumococcal isolates in the 4 regions of Hainan province

| Serotypes | | No. of isolates in each region | | | |  |
| --- | --- | --- | --- | --- | --- | --- |
|  |  | Haikou | Wanning | Qiongzhong | Baisha | Total |
| 6B | 40 | | 56 | 24 | 30 | 150 |
| 19F | 28 | | 49 | 15 | 7 | 99 |
| 6A | 21 | | 17 | 26 | 24 | 88 |
| 23F | 13 | | 15 | 5 | 12 | 45 |
| 14 | 2 | | 13 | 10 | 5 | 30 |
| 19A | 2 | | 14 | 1 | 6 | 23 |
| 18C | 1 | | 5 | 0 | 1 | 7 |
| 3 | 2 | | 2 | 1 | 1 | 6 |
| 9V | 0 | | 1 | 0 | 0 | 1 |
| 23A | 60 | | 16 | 9 | 10 | 95 |
| 34 | 14 | | 11 | 9 | 10 | 44 |
| NT | 10 | | 5 | 14 | 12 | 41 |
| Other serotypes | 30 | | 23 | 21 | 34 | 108 |
| *NT* nontypeable serotype | | | | | | |
